# 1,3-Dioxo-2,3-dihydro-1*H*-isoindol-2-yl 2,3,4-tri-*O*-acetyl-β-d-xyloside

**DOI:** 10.1107/S1600536812004369

**Published:** 2012-02-10

**Authors:** Runan Tian, Haoriqinbatu, Hongchang Liu, Xiaoming Wang, Yonghua Yang

**Affiliations:** a State Key Laboratory of Pharmaceutical Biotechnology, School of Life Sciences, Nanjing University, Nanjing 210093, People’s Republic of China; b College of Landscape Architecture, Nanjing Forestry University, Nanjing 210037, People’s Republic of China

## Abstract

The title compound, C_19_H_19_NO_10_, was obtained from the reaction of α-d-1-bromo-2,3,4-tri-*O*-acetylxylose with *N*-hy­droxy­phthalimide in the presence of potassium carbonate. The asymmetric unit contains two independent mol­ecules, in which the O—CH—O—N torsion angles are 73.0 (4) and 65.0 (4)°. The hexa­pyranosyl rings adopt chair conformations and the substituent groups are in equatorial positions. In the crystal, mol­ecules are linked by nonclassical C—H⋯O hydrogen bonds.

## Related literature

For related structures, see: Yang *et al.* (2004 ▶); Wang *et al.* (2008 ▶); Bai *et al.* (2008 ▶).
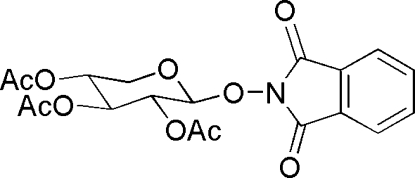


## Experimental

### 

#### Crystal data

C_19_H_19_NO_10_
*M* *_r_* = 421.35
Monoclinic, 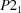

*a* = 11.722 (2) Å
*b* = 9.2270 (18) Å
*c* = 19.615 (4) Å
β = 104.52 (3)°
*V* = 2053.8 (7) Å^3^
*Z* = 4
Mo *K*α radiation
μ = 0.11 mm^−1^
*T* = 295 K
0.40 × 0.30 × 0.20 mm

#### Data collection

Enraf–Nonius CAD-4 diffractometer
Absorption correction: ψ scan (North *et al.*, 1968 ▶) *T*_min_ = 0.957, *T*_max_ = 0.978
4180 measured reflections
3977 independent reflections
2784 reflections with *I* > 2σ(*I*)
*R*_int_ = 0.058
3 standard reflections every 200 reflections intensity decay: 1%

#### Refinement

*R*[*F* ^2^ > 2σ(*F* ^2^)] = 0.047
*wR*(*F* ^2^) = 0.131
*S* = 1.00
3977 reflections
541 parameters
6 restraints
H-atom parameters constrained
Δρ_max_ = 0.24 e Å^−3^
Δρ_min_ = −0.22 e Å^−3^

### 

Data collection: *CAD-4 EXPRESS* (Enraf–Nonius, 1994 ▶); cell refinement: *CAD-4 EXPRESS*; data reduction: *XCAD4* (Harms & Wocadlo, 1995 ▶); program(s) used to solve structure: *SHELXS97* (Sheldrick, 2008 ▶); program(s) used to refine structure: *SHELXL97* (Sheldrick, 2008 ▶); molecular graphics: *ORTEP-3* (Farrugia, 1997 ▶); software used to prepare material for publication: *SHELXL97*.

## Supplementary Material

Crystal structure: contains datablock(s) global, I. DOI: 10.1107/S1600536812004369/rk2311sup1.cif

Structure factors: contains datablock(s) I. DOI: 10.1107/S1600536812004369/rk2311Isup2.hkl

Additional supplementary materials:  crystallographic information; 3D view; checkCIF report

## Figures and Tables

**Table 1:** Hydrogen-bond geometry (Å, °)

*D*—H⋯*A*	*D*—H	H⋯*A*	*D*⋯*A*	*D*—H⋯*A*
C11—H11*A*⋯O15^i^	0.98	2.42	3.339 (6)	157
C22—H22*A*⋯O1^ii^	0.96	2.39	3.329 (7)	165
C26—H26*A*⋯O3^iii^	0.98	2.54	3.385 (6)	144
C30—H30*B*⋯O5	0.97	2.56	3.429 (6)	149
C35—H35⋯O5^iv^	0.93	2.54	3.294 (8)	138

Symmetry codes: (i) 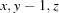; (ii) 
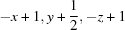
; (iii) 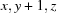; (iv) 
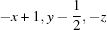
.

**Fig. 1.**
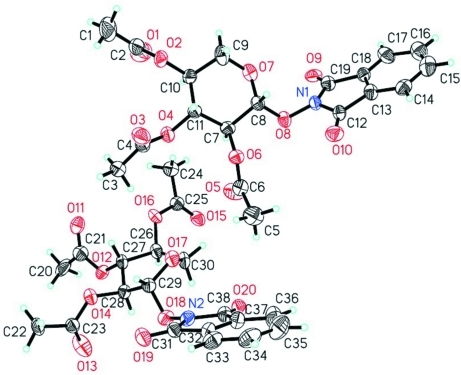
The asymmetric unit of the title compound, showing the atom-labelling scheme. Displacement ellipsoids are drawn at the 50% probability level. H atoms are presented as a small spheres of arbitrary radius.

## supplementary crystallographic information

### Comment

In the present work, the structure of 2,3,4-tri-*O*-acetyl-*β*-D-xyloside-*N*-hydroxyphthalimide, I, has been determined (Fig. 1). The asymmetric unit of I contains two independent molecules. The molecules are twisted at the CH–O bonds with the O7–C8–O8–N1 and O17–C29–O18–N2 torsion angles of 73.0 (4)° (molecule 1) and 65.0 (4)° (molecule 2), respectively. The bond lengths and angles in the title molecules show normal values. The hexapyranosyl ring adopts chair conformation (Fig. 1) and the substituented groups are individually planar and occupy equatorial positions (Yang *et al.*, 2004; Wang *et al.*, 2008; Bai *et al.*, 2008).

### Experimental

The solution of α-D-1-bromo-2,3,4-tri-*O*-acetyl-xylose (0.1 mol) and *N*-hydroxyphthalimide (0.1 mol) in chloroform (100 ml) and water (100 ml) was treated with sodium carbonate (0.1 mol) with triethyl benzyl ammonium chloride in present at room temperature overnight. The chloroform layer was separated, washed with water and allowed to evaporate slowly. The residual 2,3,4-tri-*O*-acetyl-*β*-D-xyloside-*N*- hydroxyphthalimide was then recrystallized to constant melting point (m.p. 455.6-456 K) from ethyl acetate. The purity of the compound was checked and characterized by NMR spectra. Fine block colourless crystals for X-ray diffraction were obtained by slow evaporation of an ethyl acetate at room temperature. ^1^H NMR, 500 MHz, CDCl_3_, δ: 8.15 (d, J = 9.6 Hz, 1*H*, *Ar*–H), 7.85 (d, J = 9.3 Hz, 1*H*, *Ar*–H), 5.82(d, J = 8.2 Hz, 1*H*, *G*–H), 5.25 (t, J = 9.9 Hz, 1*H*, *G*–H), 4.87(t, J = 9.6 Hz, 1*H*, *G*–H), 4.62 (t, J = 9.0 Hz, 1H, *G*–H), 3.80 (m, 2*H*, *G*–H), 2.14, 2.12, 2.09 (3s, COCH_3_).

### Refinement

Hydrogen atoms were placed in calculated positions with appropriate riding models: C–H = 0.96Å for methyl H; C–H = 0.93Å for aryl H; C–H = 0.98Å for methine H and *U*_iso_(H) = 1.2(1.5)*U*_eq_(C). The atom C25 restrictive refinement by AFIX2 command.

### Crystal data

**Table:**

C_19_H_19_NO_10_	*F*(000) = 880
*M**_r_* = 421.35	*D*_x_ = 1.363 Mg m^−^^3^
Monoclinic, *P*2_1_	Melting point = 455.6–456 K
Hall symbol: P 2yb	Mo *K*α radiation, λ = 0.71073 Å
*a* = 11.722 (2) Å	Cell parameters from 25 reflections
*b* = 9.2270 (18) Å	θ = 9–12°
*c* = 19.615 (4) Å	µ = 0.11 mm^−^^1^
β = 104.52 (3)°	*T* = 295 K
*V* = 2053.8 (7) Å^3^	Block, colourless
*Z* = 4	0.40 × 0.30 × 0.20 mm

### Data collection

**Table:**

Enraf–Nonius CAD-4 diffractometer	2784 reflections with *I* > 2σ(*I*)
Radiation source: fine-focus sealed tube	*R*_int_ = 0.058
Graphite monochromator	θ_max_ = 25.3°, θ_min_ = 1.1°
ω/2θ scans	*h* = −14→13
Absorption correction: ψ scan (North *et al.*, 1968)	*k* = 0→11
*T*_min_ = 0.957, *T*_max_ = 0.978	*l* = 0→23
4180 measured reflections	3 standard reflections every 200 reflections
3977 independent reflections	intensity decay: 1%

### Refinement

**Table:**

Refinement on *F*^2^	Primary atom site location: structure-invariant direct methods
Least-squares matrix: full	Secondary atom site location: difference Fourier map
*R*[*F*^2^ > 2σ(*F*^2^)] = 0.047	Hydrogen site location: inferred from neighbouring sites
*wR*(*F*^2^) = 0.131	H-atom parameters constrained
*S* = 1.00	*w* = 1/[σ^2^(*F*_o_^2^) + (0.0835*P*)^2^] where *P* = (*F*_o_^2^ + 2*F*_c_^2^)/3
3977 reflections	(Δ/σ)_max_ < 0.001
541 parameters	Δρ_max_ = 0.24 e Å^−^^3^
6 restraints	Δρ_min_ = −0.22 e Å^−^^3^

### Special details

**Table:**

Geometry. All s.u.'s (except the s.u. in the dihedral angle between two l.s. planes) are estimated using the full covariance matrix. The cell s.u.'s are taken into account individually in the estimation of s.u.'s in distances, angles and torsion angles; correlations between s.u.'s in cell parameters are only used when they are defined by crystal symmetry. An approximate (isotropic) treatment of cell s.u.'s is used for estimating s.u.'s involving l.s. planes.
Refinement. Refinement of *F*^2^ against ALL reflections. The weighted *R*-factor *wR* and goodness of fit *S* are based on *F*^2^, conventional *R*-factors *R* are based on *F*, with *F* set to zero for negative *F*^2^. The threshold expression of *F*^2^ > σ(*F*^2^) is used only for calculating *R*-factors(gt) *etc*. and is not relevant to the choice of reflections for refinement. *R*-factors based on *F*^2^ are statistically about twice as large as those based on *F*, and *R*-factors based on ALL data will be even larger.

### Fractional atomic coordinates and isotropic or equivalent isotropic displacement parameters (Å^2^)

**Table:**

	*x*	*y*	*z*	*U*_iso_*/*U*_eq_	
C1	0.7869 (7)	−0.0615 (11)	0.5590 (4)	0.122 (3)	
H1A	0.7057	−0.0752	0.5347	0.183*	
H1B	0.7919	−0.0293	0.6062	0.183*	
H1C	0.8286	−0.1514	0.5604	0.183*	
O1	0.8981 (5)	0.1670 (8)	0.5445 (3)	0.134 (2)	
O2	0.8245 (3)	0.0151 (4)	0.45501 (15)	0.0650 (9)	
O3	0.5499 (3)	0.0723 (5)	0.3498 (2)	0.0833 (11)	
O4	0.6896 (3)	0.2426 (3)	0.36472 (17)	0.0570 (8)	
O5	0.7255 (4)	0.4574 (4)	0.2203 (2)	0.0873 (12)	
O6	0.7291 (3)	0.2142 (3)	0.22473 (15)	0.0532 (7)	
O7	1.0122 (3)	0.1240 (4)	0.34194 (17)	0.0668 (9)	
O8	0.9724 (3)	0.2386 (3)	0.23545 (16)	0.0564 (8)	
O9	1.2187 (3)	0.2686 (4)	0.28619 (19)	0.0749 (10)	
O10	0.9277 (3)	0.0843 (5)	0.10753 (18)	0.0738 (10)	
N1	1.0553 (3)	0.1711 (4)	0.20799 (18)	0.0505 (9)	
C2	0.8407 (6)	0.0501 (10)	0.5213 (3)	0.098 (2)	
C3	0.5069 (4)	0.3108 (7)	0.3831 (3)	0.0703 (15)	
H3A	0.4296	0.2725	0.3798	0.105*	
H3B	0.5018	0.3899	0.3507	0.105*	
H3C	0.5402	0.3447	0.4302	0.105*	
C4	0.5824 (4)	0.1965 (6)	0.3658 (3)	0.0603 (12)	
C5	0.6116 (6)	0.3217 (9)	0.1216 (3)	0.097 (2)	
H5A	0.5418	0.2725	0.1262	0.146*	
H5B	0.6499	0.2650	0.0928	0.146*	
H5C	0.5906	0.4146	0.0999	0.146*	
C6	0.6941 (4)	0.3424 (6)	0.1934 (3)	0.0607 (12)	
C7	0.8168 (4)	0.2182 (5)	0.2902 (2)	0.0500 (10)	
H7A	0.8351	0.3192	0.3041	0.060*	
C8	0.9257 (4)	0.1452 (5)	0.2785 (2)	0.0501 (10)	
H8A	0.9045	0.0520	0.2546	0.060*	
C9	0.9693 (4)	0.0343 (7)	0.3882 (3)	0.0712 (15)	
H9A	1.0326	0.0122	0.4292	0.085*	
H9B	0.9409	−0.0561	0.3648	0.085*	
C10	0.8700 (4)	0.1095 (6)	0.4110 (2)	0.0560 (12)	
H10A	0.8986	0.1993	0.4363	0.067*	
C11	0.7720 (4)	0.1427 (5)	0.3462 (2)	0.0491 (10)	
H11A	0.7314	0.0530	0.3273	0.059*	
C12	1.0261 (4)	0.1045 (6)	0.1424 (2)	0.0562 (12)	
C13	1.1421 (4)	0.0666 (5)	0.1293 (2)	0.0569 (12)	
C14	1.1708 (5)	−0.0142 (6)	0.0768 (3)	0.0657 (14)	
H14	1.1125	−0.0563	0.0412	0.079*	
C15	1.2878 (6)	−0.0308 (7)	0.0784 (4)	0.0845 (17)	
H15	1.3087	−0.0842	0.0432	0.101*	
C16	1.3747 (5)	0.0298 (7)	0.1309 (3)	0.0835 (17)	
H16	1.4533	0.0196	0.1302	0.100*	
C17	1.3452 (5)	0.1074 (6)	0.1859 (3)	0.0793 (16)	
H17	1.4032	0.1463	0.2226	0.095*	
C18	1.2312 (4)	0.1231 (5)	0.1834 (2)	0.0529 (11)	
C19	1.1734 (4)	0.1982 (6)	0.2336 (2)	0.0564 (11)	
O11	0.4128 (4)	0.7342 (6)	0.4406 (2)	0.0991 (14)	
O12	0.3423 (3)	0.8357 (4)	0.33808 (15)	0.0564 (8)	
O13	0.0676 (3)	0.8442 (5)	0.2317 (3)	0.0949 (14)	
O14	0.1691 (3)	0.6402 (4)	0.26372 (16)	0.0538 (8)	
O15	0.7126 (3)	0.7988 (4)	0.29705 (18)	0.0669 (9)	
O16	0.5774 (2)	0.6731 (4)	0.33357 (16)	0.0566 (8)	
O17	0.4092 (3)	0.5377 (3)	0.19975 (17)	0.0581 (8)	
O18	0.2382 (3)	0.6188 (3)	0.12131 (16)	0.0591 (8)	
O19	0.0646 (3)	0.4089 (4)	0.07830 (19)	0.0710 (10)	
O20	0.3982 (3)	0.5904 (5)	0.03530 (19)	0.0799 (11)	
N2	0.2382 (4)	0.5133 (5)	0.0720 (2)	0.0599 (10)	
C20	0.3261 (6)	0.9705 (9)	0.4350 (3)	0.104 (2)	
H20A	0.3465	0.9642	0.4854	0.156*	
H20B	0.2427	0.9845	0.4180	0.156*	
H20C	0.3669	1.0507	0.4207	0.156*	
C21	0.3611 (5)	0.8328 (8)	0.4048 (3)	0.0790 (16)	
C22	−0.0176 (4)	0.6513 (7)	0.2828 (3)	0.0678 (14)	
H22A	0.0112	0.6384	0.3327	0.102*	
H22B	−0.0351	0.5585	0.2606	0.102*	
H22C	−0.0877	0.7094	0.2732	0.102*	
C23	0.0733 (4)	0.7248 (6)	0.2550 (2)	0.0569 (12)	
C24	0.7675 (5)	0.6422 (8)	0.3972 (3)	0.0842 (17)	
H24A	0.7657	0.5396	0.3891	0.126*	
H24B	0.7434	0.6620	0.4396	0.126*	
H24C	0.8462	0.6777	0.4021	0.126*	
C25	0.6864 (4)	0.7153 (6)	0.3371 (2)	0.059	
C26	0.4855 (4)	0.7379 (5)	0.2796 (2)	0.0525 (11)	
H26A	0.4995	0.8422	0.2770	0.063*	
C27	0.3697 (3)	0.7111 (5)	0.3015 (2)	0.0496 (10)	
H27A	0.3784	0.6260	0.3323	0.060*	
C28	0.2668 (4)	0.6885 (5)	0.2378 (2)	0.0473 (10)	
H28A	0.2473	0.7789	0.2112	0.057*	
C29	0.2899 (4)	0.5686 (5)	0.1910 (2)	0.0517 (11)	
H29A	0.2490	0.4806	0.1998	0.062*	
C30	0.4795 (4)	0.6667 (6)	0.2084 (2)	0.0614 (13)	
H30A	0.4458	0.7344	0.1709	0.074*	
H30B	0.5585	0.6428	0.2051	0.074*	
C31	0.1439 (4)	0.4156 (5)	0.0513 (2)	0.0523 (11)	
C32	0.1726 (4)	0.3348 (5)	−0.0062 (2)	0.0517 (11)	
C33	0.1103 (5)	0.2213 (6)	−0.0463 (2)	0.0698 (14)	
H33	0.0399	0.1875	−0.0387	0.084*	
C34	0.1565 (6)	0.1625 (7)	−0.0968 (3)	0.0941 (19)	
H34	0.1193	0.0848	−0.1235	0.113*	
C35	0.2655 (7)	0.2222 (10)	−0.1092 (4)	0.125 (3)	
H35	0.2941	0.1859	−0.1459	0.149*	
C36	0.3239 (6)	0.3267 (8)	−0.0691 (4)	0.097 (2)	
H36	0.3956	0.3599	−0.0752	0.117*	
C37	0.2751 (4)	0.3856 (6)	−0.0175 (2)	0.0610 (13)	
C38	0.3176 (4)	0.5078 (6)	0.0295 (2)	0.0577 (12)	

### Atomic displacement parameters (Å^2^)

**Table:**

	*U*^11^	*U*^22^	*U*^33^	*U*^12^	*U*^13^	*U*^23^
C1	0.140 (6)	0.152 (8)	0.090 (5)	−0.014 (6)	0.062 (4)	0.032 (5)
O1	0.155 (5)	0.141 (5)	0.104 (3)	−0.035 (4)	0.029 (3)	−0.008 (4)
O2	0.082 (2)	0.069 (2)	0.0509 (19)	−0.0061 (19)	0.0297 (16)	0.0099 (17)
O3	0.073 (2)	0.068 (3)	0.115 (3)	−0.013 (2)	0.034 (2)	−0.020 (2)
O4	0.0583 (18)	0.0449 (18)	0.0749 (19)	−0.0017 (15)	0.0299 (15)	−0.0017 (16)
O5	0.095 (3)	0.043 (2)	0.111 (3)	0.002 (2)	0.001 (2)	0.015 (2)
O6	0.0639 (18)	0.0388 (17)	0.0554 (17)	−0.0029 (15)	0.0120 (14)	0.0043 (15)
O7	0.0572 (18)	0.070 (2)	0.076 (2)	0.0014 (18)	0.0216 (16)	0.017 (2)
O8	0.0640 (18)	0.0413 (17)	0.0710 (19)	0.0000 (15)	0.0302 (16)	0.0071 (16)
O9	0.073 (2)	0.073 (3)	0.078 (2)	−0.014 (2)	0.0159 (18)	−0.028 (2)
O10	0.062 (2)	0.083 (3)	0.070 (2)	−0.003 (2)	0.0042 (18)	−0.005 (2)
N1	0.052 (2)	0.052 (2)	0.053 (2)	−0.0043 (18)	0.0235 (16)	0.0007 (18)
C2	0.095 (4)	0.144 (7)	0.057 (3)	−0.037 (5)	0.022 (3)	−0.008 (4)
C3	0.063 (3)	0.076 (4)	0.073 (3)	−0.002 (3)	0.018 (3)	−0.007 (3)
C4	0.056 (3)	0.055 (3)	0.071 (3)	−0.006 (2)	0.019 (2)	−0.007 (3)
C5	0.093 (4)	0.105 (5)	0.084 (4)	0.001 (4)	0.002 (3)	0.024 (4)
C6	0.063 (3)	0.056 (3)	0.062 (3)	0.008 (3)	0.013 (2)	0.006 (3)
C7	0.065 (3)	0.031 (2)	0.055 (2)	−0.006 (2)	0.016 (2)	0.003 (2)
C8	0.053 (2)	0.042 (2)	0.057 (2)	0.000 (2)	0.016 (2)	0.006 (2)
C9	0.066 (3)	0.082 (4)	0.070 (3)	0.020 (3)	0.026 (3)	0.030 (3)
C10	0.061 (3)	0.059 (3)	0.049 (2)	−0.006 (2)	0.016 (2)	0.000 (2)
C11	0.052 (2)	0.041 (2)	0.055 (2)	0.000 (2)	0.015 (2)	−0.004 (2)
C12	0.065 (3)	0.059 (3)	0.048 (2)	−0.012 (2)	0.020 (2)	0.006 (2)
C13	0.070 (3)	0.042 (3)	0.062 (3)	0.000 (2)	0.024 (2)	0.007 (2)
C14	0.094 (4)	0.047 (3)	0.057 (3)	0.001 (3)	0.021 (3)	−0.002 (2)
C15	0.114 (5)	0.055 (3)	0.098 (4)	0.016 (3)	0.053 (4)	−0.006 (3)
C16	0.077 (3)	0.078 (4)	0.105 (4)	0.008 (3)	0.039 (3)	−0.002 (4)
C17	0.076 (4)	0.062 (4)	0.104 (4)	−0.007 (3)	0.029 (3)	−0.001 (3)
C18	0.062 (3)	0.049 (3)	0.049 (2)	−0.002 (2)	0.018 (2)	0.011 (2)
C19	0.063 (3)	0.047 (3)	0.060 (3)	0.002 (2)	0.018 (2)	0.003 (2)
O11	0.124 (3)	0.108 (4)	0.066 (2)	0.033 (3)	0.025 (2)	0.002 (3)
O12	0.0646 (18)	0.0526 (19)	0.0551 (19)	0.0081 (16)	0.0207 (15)	−0.0096 (16)
O13	0.072 (2)	0.061 (3)	0.159 (4)	0.024 (2)	0.042 (3)	0.039 (3)
O14	0.0517 (17)	0.0447 (17)	0.0716 (19)	0.0041 (15)	0.0278 (15)	0.0066 (16)
O15	0.066 (2)	0.052 (2)	0.089 (2)	−0.0079 (17)	0.0298 (18)	0.0012 (19)
O16	0.0430 (16)	0.056 (2)	0.074 (2)	−0.0074 (15)	0.0196 (15)	0.0050 (17)
O17	0.0638 (19)	0.0404 (18)	0.076 (2)	0.0079 (15)	0.0285 (16)	−0.0060 (16)
O18	0.078 (2)	0.0381 (17)	0.0626 (19)	−0.0034 (16)	0.0193 (16)	−0.0046 (16)
O19	0.065 (2)	0.067 (2)	0.088 (2)	−0.0089 (18)	0.0313 (19)	−0.001 (2)
O20	0.084 (2)	0.080 (3)	0.087 (2)	−0.024 (2)	0.043 (2)	−0.014 (2)
N2	0.071 (3)	0.052 (2)	0.059 (2)	−0.008 (2)	0.022 (2)	−0.015 (2)
C20	0.110 (5)	0.128 (6)	0.077 (4)	0.025 (5)	0.028 (4)	−0.047 (4)
C21	0.088 (4)	0.086 (4)	0.064 (4)	0.010 (4)	0.023 (3)	−0.007 (4)
C22	0.048 (2)	0.092 (4)	0.071 (3)	0.002 (3)	0.029 (2)	0.008 (3)
C23	0.054 (3)	0.061 (3)	0.056 (3)	0.003 (3)	0.014 (2)	0.002 (3)
C24	0.072 (3)	0.099 (5)	0.080 (3)	−0.015 (4)	0.017 (3)	−0.012 (4)
C25	0.059	0.059	0.059	0.000	0.015	0.000
C26	0.057 (2)	0.039 (2)	0.071 (3)	0.004 (2)	0.032 (2)	0.002 (2)
C27	0.053 (2)	0.040 (2)	0.064 (3)	−0.001 (2)	0.028 (2)	−0.002 (2)
C28	0.058 (2)	0.035 (2)	0.054 (2)	−0.003 (2)	0.024 (2)	0.0039 (19)
C29	0.061 (3)	0.033 (2)	0.065 (3)	−0.007 (2)	0.024 (2)	−0.005 (2)
C30	0.062 (3)	0.064 (3)	0.071 (3)	0.007 (3)	0.040 (2)	0.003 (3)
C31	0.052 (2)	0.049 (3)	0.058 (3)	−0.003 (2)	0.018 (2)	0.001 (2)
C32	0.064 (3)	0.049 (3)	0.045 (2)	0.005 (2)	0.018 (2)	0.002 (2)
C33	0.098 (4)	0.048 (3)	0.059 (3)	−0.003 (3)	0.010 (3)	−0.002 (3)
C34	0.140 (5)	0.063 (4)	0.074 (4)	0.005 (4)	0.015 (4)	−0.023 (3)
C35	0.166 (7)	0.119 (7)	0.107 (5)	0.018 (6)	0.068 (5)	−0.046 (5)
C36	0.121 (5)	0.083 (5)	0.110 (5)	−0.007 (4)	0.072 (4)	−0.017 (4)
C37	0.064 (3)	0.066 (3)	0.057 (3)	0.007 (3)	0.022 (2)	0.005 (3)
C38	0.057 (3)	0.061 (3)	0.059 (3)	0.001 (3)	0.022 (2)	0.000 (3)

### Geometric parameters (Å, º)

**Table:**

C1—C2	1.497 (10)	O11—C21	1.214 (7)
C1—H1A	0.9600	O12—C21	1.272 (6)
C1—H1B	0.9600	O12—C27	1.433 (5)
C1—H1C	0.9600	O13—C23	1.188 (6)
O1—C2	1.292 (10)	O14—C23	1.343 (6)
O2—C2	1.307 (7)	O14—C28	1.435 (5)
O2—C10	1.421 (5)	O15—C25	1.194 (6)
O3—C4	1.224 (6)	O16—C25	1.321 (6)
O4—C4	1.332 (6)	O16—C26	1.437 (5)
O4—C11	1.446 (5)	O17—C29	1.395 (5)
O5—C6	1.201 (6)	O17—C30	1.433 (6)
O6—C6	1.349 (6)	O18—N2	1.373 (5)
O6—C7	1.430 (5)	O18—C29	1.426 (5)
O7—C8	1.408 (5)	O19—C31	1.180 (5)
O7—C9	1.410 (6)	O20—C38	1.198 (6)
O8—N1	1.373 (4)	N2—C38	1.397 (6)
O8—C8	1.410 (5)	N2—C31	1.405 (6)
O9—C19	1.222 (6)	C20—C21	1.501 (9)
O10—C12	1.199 (5)	C20—H20A	0.9600
N1—C19	1.372 (6)	C20—H20B	0.9600
N1—C12	1.388 (6)	C20—H20C	0.9600
C3—C4	1.471 (7)	C22—C23	1.477 (7)
C3—H3A	0.9600	C22—H22A	0.9600
C3—H3B	0.9600	C22—H22B	0.9600
C3—H3C	0.9600	C22—H22C	0.9600
C5—C6	1.508 (8)	C24—C25	1.479 (8)
C5—H5A	0.9600	C24—H24A	0.9600
C5—H5B	0.9600	C24—H24B	0.9600
C5—H5C	0.9600	C24—H24C	0.9600
C7—C11	1.502 (6)	C26—C30	1.530 (7)
C7—C8	1.511 (6)	C26—C27	1.542 (5)
C7—H7A	0.9800	C26—H26A	0.9800
C8—H8A	0.9800	C27—C28	1.517 (6)
C9—C10	1.516 (6)	C27—H27A	0.9800
C9—H9A	0.9700	C28—C29	1.506 (6)
C9—H9B	0.9700	C28—H28A	0.9800
C10—C11	1.516 (6)	C29—H29A	0.9800
C10—H10A	0.9800	C30—H30A	0.9700
C11—H11A	0.9800	C30—H30B	0.9700
C12—C13	1.488 (7)	C31—C32	1.461 (6)
C13—C14	1.381 (7)	C32—C37	1.359 (6)
C13—C18	1.390 (6)	C32—C33	1.401 (6)
C14—C15	1.372 (8)	C33—C34	1.355 (8)
C14—H14	0.9300	C33—H33	0.9300
C15—C16	1.373 (9)	C34—C35	1.466 (5)
C15—H15	0.9300	C34—H34	0.9300
C16—C17	1.407 (8)	C35—C36	1.321 (9)
C16—H16	0.9300	C35—H35	0.9300
C17—C18	1.334 (7)	C36—C37	1.392 (7)
C17—H17	0.9300	C36—H36	0.9300
C18—C19	1.497 (7)	C37—C38	1.463 (7)
			
C2—C1—H1A	109.5	C21—O12—C27	119.7 (4)
C2—C1—H1B	109.5	C23—O14—C28	119.4 (4)
H1A—C1—H1B	109.5	C25—O16—C26	116.9 (4)
C2—C1—H1C	109.5	C29—O17—C30	111.9 (3)
H1A—C1—H1C	109.5	N2—O18—C29	111.7 (3)
H1B—C1—H1C	109.5	O18—N2—C38	124.3 (4)
C2—O2—C10	118.0 (5)	O18—N2—C31	121.3 (4)
C4—O4—C11	119.5 (4)	C38—N2—C31	113.5 (4)
C6—O6—C7	117.1 (4)	C21—C20—H20A	109.5
C8—O7—C9	110.9 (3)	C21—C20—H20B	109.5
N1—O8—C8	112.1 (3)	H20A—C20—H20B	109.5
C19—N1—O8	121.5 (4)	C21—C20—H20C	109.5
C19—N1—C12	114.3 (4)	H20A—C20—H20C	109.5
O8—N1—C12	122.0 (4)	H20B—C20—H20C	109.5
O1—C2—O2	119.3 (6)	O11—C21—O12	122.9 (6)
O1—C2—C1	130.3 (6)	O11—C21—C20	123.5 (6)
O2—C2—C1	110.3 (7)	O12—C21—C20	113.2 (6)
C4—C3—H3A	109.5	C23—C22—H22A	109.5
C4—C3—H3B	109.5	C23—C22—H22B	109.5
H3A—C3—H3B	109.5	H22A—C22—H22B	109.5
C4—C3—H3C	109.5	C23—C22—H22C	109.5
H3A—C3—H3C	109.5	H22A—C22—H22C	109.5
H3B—C3—H3C	109.5	H22B—C22—H22C	109.5
O3—C4—O4	122.0 (5)	O13—C23—O14	123.5 (5)
O3—C4—C3	124.3 (5)	O13—C23—C22	126.3 (5)
O4—C4—C3	113.6 (5)	O14—C23—C22	110.1 (5)
C6—C5—H5A	109.5	C25—C24—H24A	109.5
C6—C5—H5B	109.5	C25—C24—H24B	109.5
H5A—C5—H5B	109.5	H24A—C24—H24B	109.5
C6—C5—H5C	109.5	C25—C24—H24C	109.5
H5A—C5—H5C	109.5	H24A—C24—H24C	109.5
H5B—C5—H5C	109.5	H24B—C24—H24C	109.5
O5—C6—O6	123.3 (4)	O15—C25—O16	124.1 (4)
O5—C6—C5	125.2 (5)	O15—C25—C24	126.8 (5)
O6—C6—C5	111.4 (5)	O16—C25—C24	109.1 (4)
O6—C7—C11	109.9 (3)	O16—C26—C30	110.4 (4)
O6—C7—C8	107.2 (3)	O16—C26—C27	106.2 (3)
C11—C7—C8	111.6 (4)	C30—C26—C27	110.1 (4)
O6—C7—H7A	109.4	O16—C26—H26A	110.1
C11—C7—H7A	109.4	C30—C26—H26A	110.1
C8—C7—H7A	109.4	C27—C26—H26A	110.1
O7—C8—O8	108.2 (3)	O12—C27—C28	106.9 (3)
O7—C8—C7	112.1 (3)	O12—C27—C26	109.5 (4)
O8—C8—C7	106.7 (3)	C28—C27—C26	111.5 (3)
O7—C8—H8A	109.9	O12—C27—H27A	109.6
O8—C8—H8A	109.9	C28—C27—H27A	109.6
C7—C8—H8A	109.9	C26—C27—H27A	109.6
O7—C9—C10	110.2 (4)	O14—C28—C29	105.6 (3)
O7—C9—H9A	109.6	O14—C28—C27	107.0 (3)
C10—C9—H9A	109.6	C29—C28—C27	112.1 (4)
O7—C9—H9B	109.6	O14—C28—H28A	110.7
C10—C9—H9B	109.6	C29—C28—H28A	110.7
H9A—C9—H9B	108.1	C27—C28—H28A	110.7
O2—C10—C11	108.2 (4)	O17—C29—O18	110.9 (3)
O2—C10—C9	109.4 (4)	O17—C29—C28	113.8 (4)
C11—C10—C9	108.7 (4)	O18—C29—C28	104.3 (4)
O2—C10—H10A	110.2	O17—C29—H29A	109.2
C11—C10—H10A	110.2	O18—C29—H29A	109.2
C9—C10—H10A	110.2	C28—C29—H29A	109.2
O4—C11—C7	105.4 (4)	O17—C30—C26	111.0 (3)
O4—C11—C10	109.3 (3)	O17—C30—H30A	109.4
C7—C11—C10	112.2 (4)	C26—C30—H30A	109.4
O4—C11—H11A	109.9	O17—C30—H30B	109.4
C7—C11—H11A	109.9	C26—C30—H30B	109.4
C10—C11—H11A	109.9	H30A—C30—H30B	108.0
O10—C12—N1	125.2 (4)	O19—C31—N2	123.8 (5)
O10—C12—C13	130.9 (4)	O19—C31—C32	132.8 (5)
N1—C12—C13	103.9 (4)	N2—C31—C32	103.4 (4)
C14—C13—C18	119.7 (5)	C37—C32—C33	121.6 (4)
C14—C13—C12	131.3 (5)	C37—C32—C31	109.6 (4)
C18—C13—C12	108.9 (4)	C33—C32—C31	128.9 (4)
C15—C14—C13	118.1 (5)	C34—C33—C32	117.6 (5)
C15—C14—H14	120.9	C34—C33—H33	121.2
C13—C14—H14	120.9	C32—C33—H33	121.2
C14—C15—C16	121.4 (5)	C33—C34—C35	119.6 (5)
C14—C15—H15	119.3	C33—C34—H34	120.2
C16—C15—H15	119.3	C35—C34—H34	120.2
C15—C16—C17	120.2 (5)	C36—C35—C34	121.3 (6)
C15—C16—H16	119.9	C36—C35—H35	119.4
C17—C16—H16	119.9	C34—C35—H35	119.4
C18—C17—C16	117.7 (6)	C35—C36—C37	118.1 (6)
C18—C17—H17	121.2	C35—C36—H36	120.9
C16—C17—H17	121.2	C37—C36—H36	120.9
C17—C18—C13	122.7 (5)	C32—C37—C36	121.7 (5)
C17—C18—C19	129.9 (5)	C32—C37—C38	109.7 (4)
C13—C18—C19	107.4 (4)	C36—C37—C38	128.5 (5)
O9—C19—N1	126.2 (4)	O20—C38—N2	123.3 (5)
O9—C19—C18	129.0 (4)	O20—C38—C37	133.3 (4)
N1—C19—C18	104.8 (4)	N2—C38—C37	103.4 (4)
			
C8—O8—N1—C19	−104.9 (4)	C29—O18—N2—C38	−102.4 (5)
C8—O8—N1—C12	93.0 (4)	C29—O18—N2—C31	89.3 (5)
C10—O2—C2—O1	−0.8 (9)	C27—O12—C21—O11	7.8 (9)
C10—O2—C2—C1	179.9 (5)	C27—O12—C21—C20	−179.1 (5)
C11—O4—C4—O3	−1.9 (7)	C28—O14—C23—O13	−6.3 (7)
C11—O4—C4—C3	−178.0 (4)	C28—O14—C23—C22	177.7 (4)
C7—O6—C6—O5	−6.3 (7)	C26—O16—C25—O15	−2.8 (7)
C7—O6—C6—C5	173.9 (4)	C26—O16—C25—C24	177.5 (4)
C6—O6—C7—C11	123.6 (4)	C25—O16—C26—C30	78.9 (5)
C6—O6—C7—C8	−114.9 (4)	C25—O16—C26—C27	−161.8 (4)
C9—O7—C8—O8	178.3 (4)	C21—O12—C27—C28	135.9 (5)
C9—O7—C8—C7	60.9 (5)	C21—O12—C27—C26	−103.2 (5)
N1—O8—C8—O7	73.0 (4)	O16—C26—C27—O12	95.9 (4)
N1—O8—C8—C7	−166.2 (3)	C30—C26—C27—O12	−144.7 (4)
O6—C7—C8—O7	−171.4 (3)	O16—C26—C27—C28	−146.0 (4)
C11—C7—C8—O7	−51.0 (5)	C30—C26—C27—C28	−26.6 (5)
O6—C7—C8—O8	70.3 (4)	C23—O14—C28—C29	−125.7 (4)
C11—C7—C8—O8	−169.3 (3)	C23—O14—C28—C27	114.7 (4)
C8—O7—C9—C10	−65.7 (5)	O12—C27—C28—O14	−70.1 (4)
C2—O2—C10—C11	−129.1 (5)	C26—C27—C28—O14	170.2 (4)
C2—O2—C10—C9	112.6 (6)	O12—C27—C28—C29	174.6 (3)
O7—C9—C10—O2	177.4 (4)	C26—C27—C28—C29	54.9 (5)
O7—C9—C10—C11	59.4 (6)	C30—O17—C29—O18	76.9 (4)
C4—O4—C11—C7	129.4 (4)	C30—O17—C29—C28	−40.3 (5)
C4—O4—C11—C10	−109.8 (5)	N2—O18—C29—O17	65.0 (4)
O6—C7—C11—O4	−75.7 (4)	N2—O18—C29—C28	−172.1 (3)
C8—C7—C11—O4	165.5 (3)	O14—C28—C29—O17	−137.4 (4)
O6—C7—C11—C10	165.4 (4)	C27—C28—C29—O17	−21.3 (5)
C8—C7—C11—C10	46.6 (5)	O14—C28—C29—O18	101.6 (4)
O2—C10—C11—O4	74.4 (5)	C27—C28—C29—O18	−142.3 (3)
C9—C10—C11—O4	−166.9 (4)	C29—O17—C30—C26	70.2 (5)
O2—C10—C11—C7	−169.0 (4)	O16—C26—C30—O17	84.7 (4)
C9—C10—C11—C7	−50.3 (5)	C27—C26—C30—O17	−32.2 (5)
C19—N1—C12—O10	−172.2 (5)	O18—N2—C31—O19	−6.6 (7)
O8—N1—C12—O10	−8.9 (7)	C38—N2—C31—O19	−176.1 (5)
C19—N1—C12—C13	8.5 (5)	O18—N2—C31—C32	174.0 (4)
O8—N1—C12—C13	171.7 (4)	C38—N2—C31—C32	4.4 (5)
O10—C12—C13—C14	−6.9 (9)	O19—C31—C32—C37	179.7 (5)
N1—C12—C13—C14	172.3 (5)	N2—C31—C32—C37	−0.9 (5)
O10—C12—C13—C18	175.5 (5)	O19—C31—C32—C33	−0.3 (9)
N1—C12—C13—C18	−5.2 (5)	N2—C31—C32—C33	179.0 (5)
C18—C13—C14—C15	−2.7 (7)	C37—C32—C33—C34	0.6 (7)
C12—C13—C14—C15	179.9 (5)	C31—C32—C33—C34	−179.4 (5)
C13—C14—C15—C16	0.6 (9)	C32—C33—C34—C35	−2.2 (9)
C14—C15—C16—C17	1.9 (10)	C33—C34—C35—C36	4.2 (12)
C15—C16—C17—C18	−2.1 (9)	C34—C35—C36—C37	−4.2 (12)
C16—C17—C18—C13	−0.1 (8)	C33—C32—C37—C36	−0.6 (8)
C16—C17—C18—C19	179.6 (5)	C31—C32—C37—C36	179.3 (5)
C14—C13—C18—C17	2.5 (8)	C33—C32—C37—C38	177.4 (4)
C12—C13—C18—C17	−179.6 (5)	C31—C32—C37—C38	−2.7 (6)
C14—C13—C18—C19	−177.2 (4)	C35—C36—C37—C32	2.5 (10)
C12—C13—C18—C19	0.6 (5)	C35—C36—C37—C38	−175.1 (6)
O8—N1—C19—O9	8.5 (7)	O18—N2—C38—O20	4.7 (8)
C12—N1—C19—O9	171.9 (5)	C31—N2—C38—O20	173.9 (5)
O8—N1—C19—C18	−171.5 (4)	O18—N2—C38—C37	−175.1 (4)
C12—N1—C19—C18	−8.2 (5)	C31—N2—C38—C37	−5.9 (5)
C17—C18—C19—O9	4.5 (9)	C32—C37—C38—O20	−174.7 (6)
C13—C18—C19—O9	−175.8 (5)	C36—C37—C38—O20	3.1 (10)
C17—C18—C19—N1	−175.5 (5)	C32—C37—C38—N2	5.1 (5)
C13—C18—C19—N1	4.2 (5)	C36—C37—C38—N2	−177.1 (6)

### Hydrogen-bond geometry (Å, º)

**Table:**

*D*—H···*A*	*D*—H	H···*A*	*D*···*A*	*D*—H···*A*
C7—H7*A*···O5	0.98	2.22	2.676 (6)	107
C10—H10*A*···O1	0.98	2.14	2.609 (7)	107
C11—H11*A*···O3	0.98	2.29	2.702 (6)	104
C11—H11*A*···O15^i^	0.98	2.42	3.339 (6)	157
C22—H22*A*···O1^ii^	0.96	2.39	3.329 (7)	165
C26—H26*A*···O3^iii^	0.98	2.54	3.385 (6)	144
C27—H27*A*···O11	0.98	2.29	2.656 (6)	101
C28—H28*A*···O13	0.98	2.32	2.718 (6)	103
C30—H30*B*···O5	0.97	2.56	3.429 (6)	149
C35—H35···O5^iv^	0.93	2.54	3.294 (8)	138

Symmetry codes: (i) *x*, *y*−1, *z*; (ii) −*x*+1, *y*+1/2, −*z*+1; (iii) *x*, *y*+1, *z*; (iv) −*x*+1, *y*−1/2, −*z*.
